# Upper Critical Solution Temperature (UCST) Behavior of Coacervate of Cationic Protamine and Multivalent Anions

**DOI:** 10.3390/polym11040691

**Published:** 2019-04-16

**Authors:** Hyungbin Kim, Byoung-jin Jeon, Sangsik Kim, YongSeok Jho, Dong Soo Hwang

**Affiliations:** 1 Division of Integrative Biosciences and Biotechnology, Pohang University of Science and Technology (POSTECH), Pohang 37673, Korea; hbkim22@postech.ac.kr; 2 Materials Department, University of California, Santa Barbara, Santa Barbara, CA 93106, USA; bjeon8@gmail.com; 3 Division of Environmental Science and Engineering, Pohang University of Science and Technology (POSTECH), Pohang 37673, Korea; ukssloveu@postech.ac.kr; 4 Department of Physics and Research Institute of Natural Science, Gyeongsang National University, Jinju 52828, Korea

**Keywords:** coacervate, UCST, multivalent ion, protamine, guanidine, temperature

## Abstract

Complex coacervation is an emerging liquid/liquid phase separation (LLPS) phenomenon that behaves as a membrane-less organelle in living cells. Yet while one of the critical factors for complex coacervation is temperature, little analysis and research has been devoted to the temperature effect on complex coacervation. Here, we performed a complex coacervation of cationic protamine and multivalent anions (citrate and tripolyphosphate (TPP)). Both mixtures (i.e., protamine/citrate and protamine/TPP) underwent coacervation in an aqueous solution, while a mixture of protamine and sodium chloride did not. Interestingly, the complex coacervation of protamine and multivalent anions showed upper critical solution temperature (UCST) behavior, and the coacervation of protamine and multivalent anions was reversible with solution temperature changes. The large asymmetry in molecular weight between positively charged protamine (~4 kDa) and the multivalent anions (<0.4 kDa) and strong electrostatic interactions between positively charged guanidine residues in protamine and multivalent anions were likely to contribute to UCST behavior in this coacervation system.

## 1. Introduction

Coacervation is a liquid/liquid phase separation (LLPS) phenomenon in aqueous solution caused by the complexation of dissolved polymers in the aqueous solution due to a variety of attractive forces [1,2,3,4]. When an aqueous solution is adjusted to a specific pH and ionic strength at which solvated polymers attract each other, the polymer chains mingle, partially desolvate, and recruit other polymer chains to form dynamic dense polymer droplets within the fluid [1,5]. This dense dynamic polymer droplet is called a coacervate, originating from the Latin word coacervatus, which means “cluster”. The variety of attractive forces for coacervation could be electrostatic bonds, hydrogen bonds, cation-π interactions, hydrophobic interactions, and other attractive van der Waals forces in aqueous solution [6,7,8,9]. A coacervate was first reported by the Dutch scientists Bungenberg de Jong and Kruyt as an LLPS resulting from mixing two oppositely charged polyelectrolytes, a positively charged gelatin and a negatively charged gum Arabic [1].

When coacervation is driven by electrostatic attraction in water, the process is called “complex coacervation” [3]. Complex coacervates, dense polymer-rich liquid droplets, generally have a very low interfacial energy [2,10,11]. Therefore, complex coacervates can grow dynamically by coalescence from micro- to mesodroplets and eventually separate to bulk phase from aqueous solution. The low interfacial energy of complex coacervates enables the coacervates to encapsulate a variety of substances in solution, including dyes, particles, fragrances, cells, and even explosives [12,13,14,15,16].

The formation of complex coacervates is controlled by the pH and ionic strength of the solution, the type of polyelectrolyte, the concentration of the polymers, the molecular weight and conformation of the polymers, the mixing ratio between two oppositely charged polymers, and the temperature. Among the aforementioned conditions, the effect of temperature on the formation of complex coacervates has not yet been systematically studied.

**Figure 1:**
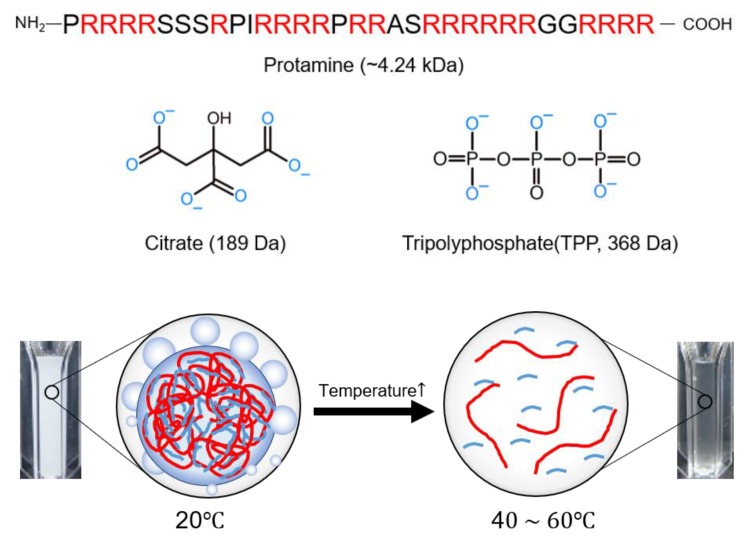
Schematic of the upper critical solution temperature (UCST) behavior of protamine/multivalent ion complex coacervation.

Contrary to the fact that conventional theories of complex coacervates based on Flory–Huggins theory [17,18], such as the Tainaka [19] or Voorn–Overbeek theories [20], predict that attractive electrostatic interaction induces upper critical solution temperature (UCST) behavior, most complex coacervates observed in experiments have shown lower critical solution temperature (LCST) behavior [21,22,23,24]. This implies that not only direct electrostatic attraction but also indirect electrostatic effects, such as charge renormalization and consequent shape change, or nonelectrostatic interactions are crucial to the formation of conventional complex coacervates. Recently, UCST behavior was reported in the complex coacervation of positively charged mussel foot protein type 3A (mfp-3A), which is rich in arginine and multivalent anions (citrate) [25]. In this study, we used protamine from salmon as a positively charged polyelectrolyte because 65% of the amino acids in the protamine primary sequence are arginine (Figure 1). As multivalent anions, we used citrate and tripolyphosphate (TPP) (Figure 1). The coacervation systems were explored with respect to the protamine/multivalent ion ratio, total polyelectrolyte concentration, pH, and temperature. Additionally, the viscosity and interfacial tension of the dense phase in LLPS during protamine/citrate coacervation were measured through microrheology and the coalescence of coacervate droplets.

## 2. Materials and Methods

### 2.1. Materials

Protamine sulfate salt from salmon (p4020), sodium citrate tribasic dehydrate (S4641), sodium tripolyphosphate (7758-29-4)and Sigmacote (SL2) were purchased from Sigma (Saint Louis, MO, USA). FITC (5(6)-SFX (6-(Fluorescein-5-(and-6)-Carboxamido) Hexanoic Acid, Succinimidyl Ester), F2181) and particles with a size of 0.2 μm (FluoSpheres) from Thermo Fisher Scientific (Waltham, MA, USA) and 2% PFPE (Perfluorinated polyethers) –PEG (Polyethyleneglycol) –PFPE (Perfluorinated polyethers) triblock copolymer surfactant (E2K0660) from RAN Biotechnologies, Inc. (Beverly, MA, USA), were obtained. Circular dichroism (CD) spectropolarimeter (J-1500, JASCO, Easton, MD, USA), Confocal microscopy (visitech, Sunderland, UK), Optical microscopy (BX63, Olympus, Japan), Zetasizer (Malvern instruments, Malvern, UK), MATLAB software (Mathworks, MA, USA) and UV-Vis spectrometer (Optizen, Gyeonggi, Korea) were used.

### 2.2. Relative Turbidity and Zeta Potential Measurements

By varying the ratio between protamine and multivalent ions (0 to 1), total polyelectrolyte concentration (*C*_p_) (0.1 to 1.6% (*w*/*v*)), and pH, coacervation of the protamine and multivalent ions was quantified by turbidity at room temperature. The solution pH values of the protamine/citrate system and protamine/TPP system were ~8.0 and ~4.5, respectively, where both multivalent ions lost three protons. The protamine and multivalent ions were dissolved in water to 1% (*w*/*v*) as stock solution and filtered with a 0.45-μm filter. The pH of sodium citrate stock (1% *w*/*v*) was titrated to ~8.0, and the pH of sodium tripolyphosphate was adjusted to ~4.5 by adding 10 mM HCl. Each stock solution was diluted to make different total polyelectrolyte concentrations (*C*_p_) and ratios between protamine and multivalent ions. Relative turbidity measurements were performed by UV–VIS spectrometer (Optizen, Gyeonggi, South Korea) at 600 nm, at which the absorbance interference from protamine was negligible. The relative turbidity was defined as -ln (*T*/*T*_0_), where *T* and *T*_0_ are the light transmittance with and without polyelectrolytes, respectively [16]. Each experiment was performed in triplicate. The formation of coacervate droplets was monitored by optical microscopy (BX63, Olympus, Japan).

### 2.3. Temperature-Dependent Turbidity Measurements

Temperature-dependent turbidity measurements were performed with a circular dichroism (CD) spectropolarimeter (J-1500, JASCO, Easton, MD, USA) with a temperature controller. The absorbance of the coacervates was obtained at 600 nm with a temperature change rate of 1 °C /min, and the chamber was purged with nitrogen gas. Each temperature cycling experiment was performed from 25 to 60 °C. The weight ratio between protamine and multivalent ions was fixed at a ratio of 6:4, and *C*_p_ was fixed at 1% (*w*/*v*).

### 2.4. Microrheological Analysis of Viscosity

The viscosity of the protamine/citrate coacervate was measured by microrheology. The dense phase of the protamine/citrate complex coacervate at a ratio of 6:4 in 1% (*w*/*v*) was prepared freshly. To determine the viscosity, microrheology was performed by embedding fluorescent probe particles (200 nm diameter, 540 ex/560 em) into the dense phase of the complex coacervate. The particle-dispersed dense phase was introduced into a flow cell, which was a channel that was formed in a sandwiched coverslip–parafilm–coverslip configuration using end-cut pipette tips and subsequently sealed with epoxy [25]. The mobility of the particles (*n* = 189) in the dense phase was tracked using confocal microscopy (Visitech, Sunderland, UK) for 50 s with 10-ms intervals at 20 °C. The averaged mean squared displacement (MSD) of the particles was calculated using MATLAB software (Mathworks, MA, USA) and fit to the form MSD(τ) ~4*D*_probe_*τ^α^*, where α is the diffusive exponent, to estimate the diffusion coefficient *D*_probe_. The viscosity η of the dense phase was calculated using the Stokes–Einstein equation, *D*_probe_ = *k_B_T*/6πη*r*, where *k_B_* is the Boltzmann constant, *T* = 293 K is temperature, and *r* = 100 nm is the probe radius.

### 2.5. Coalescence Experiments

The interfacial tension of the protamine/citrate coacervate was measured by observing coalescence events of two droplets over time. A solution containing droplets of an FITC (Fluorescein isothiocyanate)-tagged protamine/citrate coacervate phase was prepared and flowed into a coverslip-sandwiched fluid chamber with a flat oil/water interface [26] to minimize the friction from the surface during droplet coalescence. The inner surface of the bottom coverslip was made hydrophobic by treatment with Sigmacote, and the interface between the oil (3M^TM^ Novec^TM^ 7500 Engineered Fluid) and the aqueous phases was stabilized with PFPE-PEG-PFPE triblock copolymer surfactant (E2K0660, RAN Biotechnologies, Inc.). Coalescence events were recorded with confocal microscopy (Visitech) with 4-ms intervals at 488 nm excitation and decay timescales τ. Changes in the dimensionless parameter A=(L−W)/(L+W), a ratio of the difference and sum of the length (*L*) and width (*W*) of a droplet during relaxation, were measured at late stages when all coalescing droplets looked convex. We then used a formula for the relaxation kinetics of a deformed liquid droplet [27],

(1) $$\tau ≅\frac{19}{20}\frac{\eta R}{\sigma }$$

which shows the relaxation decay time, τ, as a function of viscosity (η), interfacial tension (σ), and the radius (*r*) of the liquid droplet from the Equation (1) [27].

## 3. Results

### 3.1. Complex Coacervation of Protamine and Multivalent Ions

**Figure 2:**
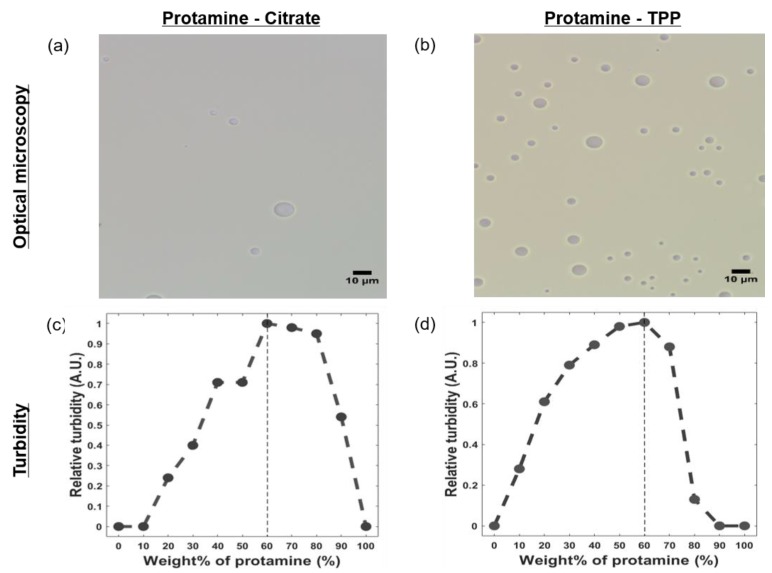
Optical microscopy image of (**a**) protamine/citrate coacervate obtained at pH 8.0 and (**b**) protamine/ tripolyphosphate (TPP) coacervate at pH 4.5. The total polyelectrolyte concentration (*C*_p_) was 1% (*w*/*v*), and the scale bar is 10 μm. The optical turbidity of (**c**) protamine/citrate coacervates and (**d**) protamine/TPP coacervates with different weight ratios. *C*_p_ was 0.1% (*w*/*v*).

Protamine (~4 kDa) from salmon sperm was selected as a positively charged polyelectrolyte, and sodium citrate (189 Da) or sodium tripolyphosphate (TPP, 368 Da) was selected as a multivalent anion (Figure 1). Protamine and one of the multivalent ions were mixed to generate a complex coacervate. By varying the weight ratio between protamine and the multivalent ions (0 to 1), complex coacervates were formed. Coacervation-dependent LLPS was observed by light microscopy and by the increase in relative turbidity of the solution. The solution pH values of the protamine/citrate and protamine/TPP systems were ~8.0 and ~4.5, respectively, where both multivalent ions lost three protons in the given solution pH. When protamine and the multivalent ions were mixed in water, the mixed solutions became turbid, and the formation of spherical coacervate droplets in both systems was observed by an optical microscope (Figure 2). The calculated positive/negative charge ratio was close to 1:1. At a protamine/multivalent ion ratio of 6:4, both systems had a maximum coacervate yield (Figure 2). Therefore, protamine/multivalent ion coacervates were prepared by mixing protamine and multivalent ion solution at a ratio of 6:4 for further experiments. As a control experiment, mixing of protamine and sodium chloride was performed, but coacervation was not observed with various protamine/sodium chloride ratios (Figure S1). This means that multivalent ions were likely to contribute to the complex coacervation of cationic protamine with anionic small molecules.

### 3.2. The Influence of Temperature on the Complex Coacervation of Protamine and Multivalent Ions

**Figure 3:**
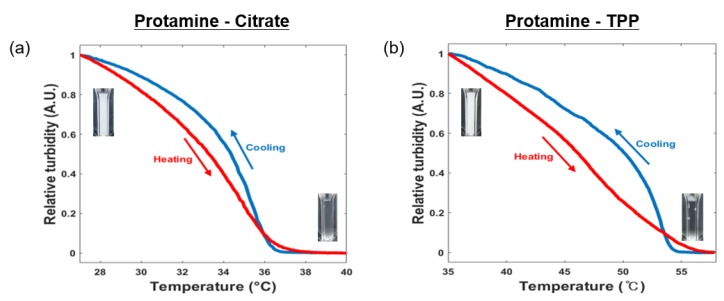
Temperature-dependent coacervation of protamine and multivalent ion mixtures. Turbidity measurements of the (**a**) protamine/citrate mixture and (**b**) protamine/TPP mixture at 600 nm with respect to temperature. The total polyelectrolyte concentration (*C*_p_) was fixed to 1% (*w*/*v*), and the weight ratio between protamine and multivalent ions was fixed to 6:4. Inset images show reversible phase transitions of the coacervates by temperature cycling.

However, while one of the critical factors for complex coacervation is temperature, little analysis and research has been devoted to the temperature effect on complex coacervation. The temperature dependence of the protamine/multivalent ion system was determined at a protamine/multivalent ion ratio of 6:4. Upon heating, the turbid mixture of protamine and multivalent ions changed to a transparent solution (red curve, Figure 3). The transparent solution became turbid following cooling to room temperature (blue curve, Figure 3). The critical solution temperature (*T*_c_) for protamine/citrate and protamine/TPP was ~33 and ~45 °C, respectively. At higher temperatures, mixing entropy dominated and favored a macroscopic homogeneous mixture.

### 3.3. The Influence of the Total Polyelectrolyte Concentration (C_p_) of the Mixture of Protamine and Multivalent Ions

**Figure 4:**
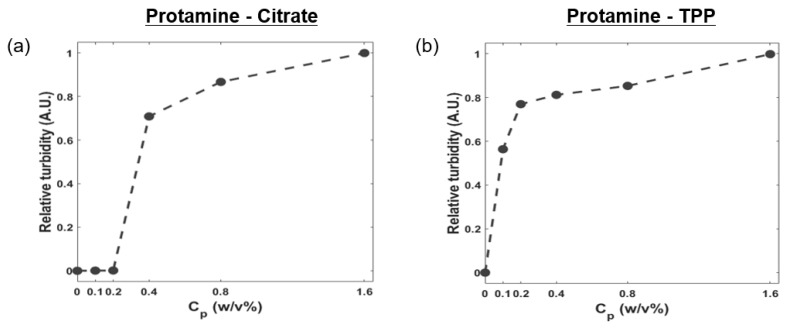
Influence of total polyelectrolyte concentration on complex coacervates. (**a**) Protamine/citrate mixture at pH 8.0 and (**b**) protamine/TPP mixture at pH 4.5. The weight ratio between protamine and multivalent ions was fixed to 6:4.

Coacervation can be affected by the total polyelectrolyte concentration (*C*_p_). *C*_p_ was defined as the weight sum of protamine and multivalent ions in the solution. The results of relative turbidity measurements for mixtures of protamine/multivalent ions with different *C*_p_ values are presented in Figure 4. As *C*_p_ in the system increased from 0.1% (*w*/*v*) to 1.6% (*w*/*v*), the relative turbidity increased in both systems. The protamine/TPP system had a higher relative turbidity when *C*_p_ was ~0.2% (*w*/*v*), implying that the bonding between positively charged guanidine in protamine and negatively charged phosphate in TPP was stronger than the bonding between positively charged guanidine in protamine and negatively charged carboxyl groups in citrate.

### 3.4. Effect of pH on Complex Coacervates of Protamine and Multivalent Ions

**Figure 5:**
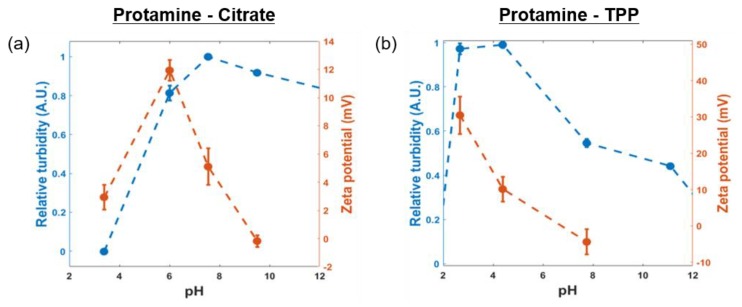
Effect of pH on complex coacervates of protamine and multivalent ions: pH-dependent turbidity and zeta potential measurements of the (**a**) protamine/citrate coacervate and (**b**) protamine/TPP coacervate.

The surface charge and the relative turbidity were measured in a pH range from ~2 to ~12. Both coacervate systems reached maximum turbidity when the surface charge determined by the Zetasizer converged to zero (Figure 5). With a protamine positive charge/multivalent ion negative charge ratio of 1:1, the maximum relative turbidity was observed, and the surface charge dropped to zero. Citrate has three carboxyl groups with three pK_a_ values of ~3.1 (pK_a1_), ~4.8 (pK_a2_), and ~6.4 (pK_a3_) at 25 °C; while TPP is a pentabasic acid with five pK_a_ values of ~1.0 (pK_a1_), ~2.2 (pK_a2_), ~2.30 (pK_a3_), ~6.50 (pK_a4_), and ~9.24 (pK_a5_); and guanidine groups in arginine have a pKa value of ~12.5. Therefore, the protamine/citrate system had maximum turbidity at pH ~8.0. In the case of TPP, the maximum turbidity was observed at pH ~4.5, where the surface charges of the complex coacervates dropped to zero. Since TPP is a pentabasic acid, the turbidity of the protamine/TPP complex decreased as the pH changed from 4.5 to 12 due to deprotonation in TPP.

### 3.5. Viscosity and Interfacial Tension of Protamine-Citrate Coacervates

**Figure 6:**
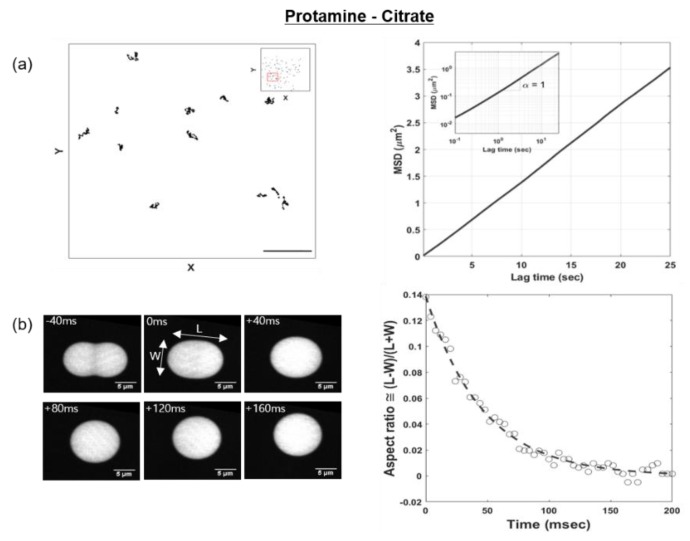
Measurements of viscosity and interfacial tension of the protamine/citrate complex coacervate. (**a**) Left: Tracked movements (*n* = 189) of fluorescent probe particles dispersed in the dense phase of the complex coacervate. Scale bar: 5 μm. Right: The averaged mean square displacement (MSD)–lag time plot of particles (0.2 µm) in the dense phase. Inset is a log–log plot. (**b**) Left: Confocal image of the coalescence of droplets. Right: Aspect ratio–time plot. The aspect ratio is defined as (*L* − *W*)/(*L* + *W*), where *L* and *W* are the length and width, respectively, of the deformed droplet under relaxation. All experiments were performed at 20 °C.

In the microrheology experiments, we observed the liquid nature of the protamine-citrate coacervate phase from α≅1 in <*MSD*> $\sim \tau^{\alpha }$ to determine the thermal fluctuation of the probe particles distributed in the coacervate phase, as shown in Figure 6a. The diffusion coefficient of the particles was found to be 0.0326 μm^2^/s from <*MSD*> = 4*D_probe_τ^α^*, and the viscosity of the coacervate phase calculated from the Stokes–Einstein equation was 0.0659 Pa·s when *T* = 293 K and *r* = 100 nm were used. We then used the measured viscosity value to extract the interfacial tension of the coacervate phase by monitoring coalescence events between two droplets, as coalescences are driven by minimizing the interfacial area and are resisted by the viscosity of the liquid phase. For each of 11 droplet coalescence events, the timescales of the deformed droplets being relaxed into spheres were extracted as described in the “Materials and Methods” section (Table S1). The interfacial tension of the protamine-citrate coacervate phases was calculated to be $7.35\times 10^{-6}\;N/m$ from Equation (1), as shown in Figure S2. The viscosity and interfacial tension of the previously reported coacervate systems varied with the pH and ionic strength of the solution, the type of polyelectrolyte, the types of intermolecular attractions, the concentration of the polymers, the molecular weight and conformation of the polymers, the mixing ratio, and the temperature. However, it should be noted that the measured viscosity and interfacial tension of the protamine-citrate system were relatively low compared to previously studied coacervate systems [1,2,3,6,7,10,11,16,22,28,29,30,31].

## 4. Discussion

A conventional complex coacervate is formed from the demixing of a polyelectrolyte complex, which is a pair of anionic and cationic polyelectrolytes and water. This kind of phase transition is generally driven by entropy, and thus LCST behavior is observed when the phase separation disappears when the temperature is lowered. On the other hand, when a relatively long positively charged polymer (protamine, 4 kDa) is crosslinked by multivalent anions (<0.4 kDa) to form a network structure, the network structure in this system is similar to that of a solid-like hydrogel. In this case, the origin of the phase transition is enthalpic. The cross-linking due to electrostatic attraction weakens with increasing temperature, and the system shows UCST behavior (Figure 3).

Interestingly, the complex coacervate found in this study was formed from strong electrical interactions, as are hydrogels, but underwent liquid–liquid phase separation, similar to conventional complex coacervates. The complex coacervate did not undergo phase separation with monovalent salts (Figure S1), but it did with multivalent anions such as citrate or TPP (Figure 2). In cases where coacervation is formed by monovalent ions, they may show LCST behavior [32]. With LCST, less solvation is expected with increasing temperature. The effective interaction may be obtained by considering the interactions among the hydrated polymer and ions, which can have nonmonotonic behavior with temperature. In our system, the electrostatic bridge mediated by multivalent anions was so strong that it did not show nonmonotonic behavior with temperature. When the concentration of multivalent anions exceeded the critical salt concentration, the multivalent anions led to macroscopic condensation by electrostatically bridging the association of oppositely charged polyelectrolytes. This polymer-rich condensate grew until it reached thermodynamic equilibrium (or at least quasi-equilibrium) with a dilute supernatant phase outside the condensate [33]. In this phase separation, the strong charge bridging effect that caused phase separation was purely electrostatic, and hence UCST behavior was observed.

In addition to temperature, phase separation depends on the valence of the multivalent anion, its concentration, pH, and hydration effects. When the valence of the multivalent anion is high, phase separation is induced even at lower concentrations, as observed in the pH control experiment, because the charge bridging becomes stronger with a higher valence of multivalent anions. However, if the pH is too high, the condensation effect of the anion is reduced, and the phase separation disappears. Another notable point is that TPP induces a phase transition at lower salt concentrations and has a higher critical solution temperature than citrate. The difference between citrate and TPP is likely to originate from the hydration effect. TPP may have a more fragile hydration shell than citrate, which can mediate a stronger electrostatic bridge [34]. The valences of citrate and TPP are the same, but because chaotropic ions form a stronger charge bridge, TPP induces complex coacervation at lower concentrations and has higher *T*_c_ than citrate.

## 5. Conclusions

In this report, the complex coacervation of positively charged protamine and multivalent anions (citrate and TPP) was studied. Unlike conventional complex coacervates made of two oppositely charged polyelectrolytes with similar molecular weights showing LCST behavior, a complex coacervate of protamine and multivalent anions showed upper critical solution temperature (UCST) behavior, which was reversible with temperature cycling. Due to the large molecular weight asymmetry in between positively charged polyelectrolytes (~4 kDa) and multivalent anions (<0.4 kDa), multivalent anions seemed to behave as crosslinkers of relatively long positively charged polymers. The electrostatic origin crosslinking was not strong enough to make a gel phase: Instead, it formed a dense liquid phase, leaving dilute supernatant outside. Therefore, this enthalpic origin phase transition showed UCST behavior. 

## Supplementary Materials

The following are available online at https://www.mdpi.com/2073-4360/11/4/691/s1, Figure S1: Characterization of turbidity of protamine/sodium chloride solution. Figure S2: Measurement of τ with variable droplet radius to determine the interfacial tension of the complex coacervate. Table S1: Measurement of τ with variable droplet radius to determine the interfacial tension of the complex coacervate.
