# Supplementary material for: Upper Critical Solution Temperature (UCST) Behavior of Coacervate of Cationic Protamine and Multivalent Anions
Source: Polymers (Basel). 2019 Apr 16;11(4):691. doi: 10.3390/polym11040691 (PMC6523134; doi:10.3390/polym11040691)
Supplement: Supplementary file 1 [file polymers-11-00691-s001.pdf]

Supplementary Information

Upper cirtical solution temperature (UCST) behavior of coacervate cationic protamine and anionic macroions

Hyungbin Kim[1], Byoung-jin Jeon[2], Sangsik Kim[1,3], YongSeok Jho [4,*] and Dong Soo Hwang [1,3,*]

1 Division of Integrative Biosciences and Biotechnology, Pohang University of Science and Technology (POSTECH), Pohang 37673, Republic of Korea ; hbkim22@postech.ac.kr
2 Materials Department, University of California, Santa Barbara, Santa Barbara, CA 93106 USA ; saleh@ucsb.edu
3 Division of Environmental Science and Engineering, Pohang University of Science and Technology (POSTECH), Pohang 37673, Republic of Korea ; uksslovevu@postech.ac.kr
4 Department of Physics and Research Institute of Natural Science, Gyeongsang National University, Jinju 52828, Republic of Korea ; ysjho@gnu.ac.kr

* Correspondence: yongseokjho@gmail.com or dshwang@postech.ac.kr; Tel.: +82-54-279-9505

Received: date; Accepted: date; Published: date

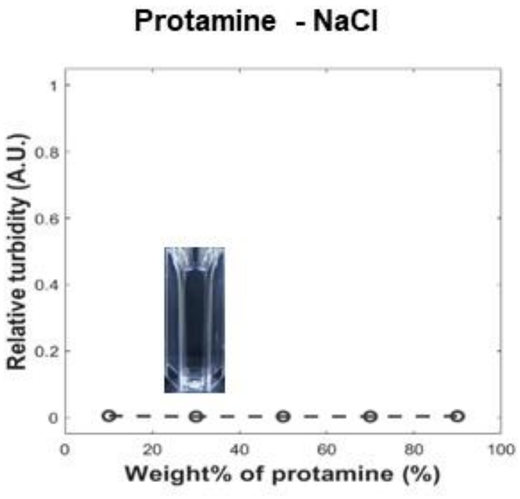

Figure S1. Turbidity of protamine/sodium chloride solution.

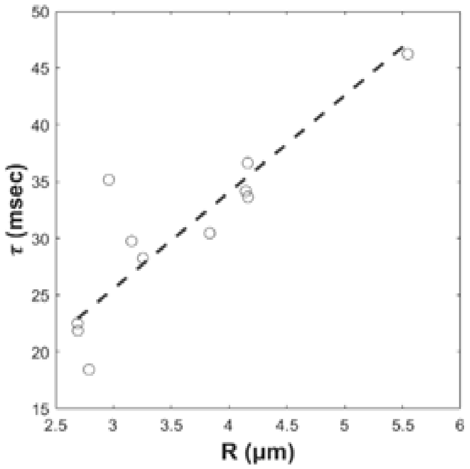

Figure S2. Measurement of τ with variable radius of droplets for interfacial tension of the complex coacervate.

FOV	R (um)	t(msec)
1	3.1570	29.74
2	miss	miss
3	2.6923	21.86
4	2.7886	18.43
5	2.9603	35.15
6	4.1624	33.63
7	2.6896	22.51
8	3.2550	28.24
9	3.8314	30.44
10	4.1602	36.63
11	5.5445	46.23
12	4.1438	34.15

Table S1. Measurement of τ with variable radius of droplets for interfacial tension of the complex coacervate.
